# Genome-Wide Study of the UK Biobank Highlights the Importance of the Homeobox-C Gene Cluster in Hip Fracture Risk

**DOI:** 10.1177/21514593251336568

**Published:** 2025-04-16

**Authors:** Louis John Koizia, Matteo Di Giovannantonio, Ping Zhang, Michael Barry Fertleman, Benjamin Howell Lole Harris

**Affiliations:** 1 Cutrale Perioperative and Ageing Research Group, 4615 Imperial College London, London, UK; 2 Department of Oncology, 6396 University of Oxford, Oxford, UK; 3 Centre for Human Genetics, 6396 University of Oxford, Oxford, UK

**Keywords:** UK Biobank, GWAS, hip fracture, HOX-C gene cluster, HOXC6, HOXC8, HOXC9, HOXC-AS1, rs4142680, rs11614913, neck of femur

## Abstract

**Introduction:**

Hip fractures are among the most common major orthopaedic injuries globally, with one in three women and one in twelve men projected to sustain a hip fracture in their lifetime. Identifying genetic factors that contribute to hip fracture risk could improve risk stratification and inform prevention strategies. This study aims to identify genetic variants associated with hip fracture susceptibility through a genome-wide association study (GWAS).

**Materials and Methods:**

A GWAS was undertaken using the UK Biobank to identify risk loci for hip fractures.

**Results:**

At the time of analysis, 2165 neck of femur fractures were identified among the 502 507 participants. Thirteen SNPs in five putative haplotypes were identified as significantly associated with hip fracture using the stringent GWAS threshold of 5E-8. Two of these loci appear to affect HOXC8, either by influencing the 3’ UTR (rs4142680[T]) or via the miRNA hsa-miR-196a (rs11614913[T]). These two SNPs were also found to be expression quantitative trait loci for homeobox-C cluster genes (HOXC6, HOXC9, and HOXC-AS1).

**Conclusions:**

Polymorphisms affecting homeobox-C cluster genes influence hip fracture risk in the general population. Future research should focus on validating these genetic associations and exploring optimal therapeutic interventions that could mitigate fracture risk in subpopulations carrying these polymorphisms.

## Introduction

Hip fractures are among the most common major orthopaedic injuries globally, with one in three women and one in twelve men projected to sustain a hip fracture in their lifetime.^1^ The number of hip fractures is expected to reach approximately 4.5 million by the year 2050.^2^ Although primarily a condition affecting the elderly, hip fractures are life-changing events associated with negative outcomes such as disability, cognitive impairment and increased dependence. The secondary care costs associated with these injuries in the UK alone are estimated at £1.1 billion annually.^3^ Readmission significantly contributes to healthcare costs, with 5%-12% of hip fracture patients discharged to post-acute care facilities being readmitted within six weeks.^4^ Furthermore, it should be noted that around 50% of patients who lived independently before sustaining a hip fracture never regain their independence.^5,6^

The significant mortality associated with a neck of femur fracture is not fully captured by headline costs to health systems. Older adults have a 5- to 8-fold increased risk for all-cause mortality during the first 3 months after hip fracture.^7^ Despite advances in perioperative geriatric care, one third of adults who have a hip fracture die within 12 months, with an increased risk of death persisting for at least 10 years.^8^ Common causes of death include deconditioning, pneumonia, ischaemic heart disease and frailty resulting from the event.^9^ Therefore, it is imperative to identify and address risk factors for this significant condition for individuals and society as a whole.

Genome-wide association studies (GWAS) are genetic studies that focus on identifying genetic variations associated with particular traits or diseases across the entire genome. GWAS analyse genetic variations across the entire genome to identify associations between specific genetic variants and a specific outcome. These variants, typically single nucleotide polymorphisms (SNPs), are tested for their frequency and distribution among individuals with the outcome and without.^10^ In the past decade, GWAS have investigated a variety of variables, with such work significantly enhancing our understanding of cancer types and anthropometric traits.^11^ GWAS also hold potential for advancing our biological understanding of hip fractures. A previous study examining hip geometry identified associations between several genes and hip structural measures, explaining 12% to 22% of heritability at different sites.^12^ However, GWAS in this field are still rare, highlighting the need for further research to uncover additional genetic determinants of fracture risk and bone morphology.

Here we independently interrogate one of the largest cohort studies in existence, the UK Biobank to identify and reaffirm SNPs associated with hip fracture risk. The UK Biobank is a large-scale biomedical database and offers a resource for investigating the intricate interplay between genetics, lifestyle factors, and health outcomes. This comprehensive resource presents an exciting opportunity to investigate if relationships exist between genetic polymorphisms and hip fractures.

## Methods

### UK Biobank

The UK Biobank is a large-scale biomedical database and research resource that collects and shares health data to support scientific research aimed at improving the prevention, diagnosis, and treatment of a wide range of diseases. Established in 2006, it is one of the most comprehensive and ambitious studies of its kind in the world.

The UK Biobank consists of approximately 500 000 UK residents who consented to share their clinical, lifestyle, anthropometric, and genetic data for research purposes. Participants were between the ages of 40 and 69 at the time of enrolment into the UK Biobank cohort study. Initially on enrolment participants underwent face-to-face interviews and sample collection. At the start they underwent a wide range of physical measures, provided information on their lifestyle and medical history, donated blood, urine and saliva samples for future analysis and agreed to have their health followed up through linkage to their health-related records. Over the subsequent 18 years, patient information was sought from inpatient hospital records, primary care data, and centralised clinical registries, including the Cancer and Death registers. Hip fracture occurrence was part of these data (mean age of occurrence is included in the results). The UK Biobank secured informed consent from all individuals, and all study protocols received approval from the National Research Ethics Service Committee. This research will be performed as per an approved UK Biobank application (number 79840) and in compliance with the Declaration of Helsinki.^13^

To obtain genetic data, blood samples were taken at the time of participant enrolment, and DNA was subsequently extracted.^14^ This DNA underwent genotyping using either the Affymetrix UK BiLEVE Axiom array or the Affymetrix UK Biobank Axiom array. The imputation process utilised a combined reference panel consisting of approximately 90 million biallelic variants derived from the 1000 Genomes Phase 3 and the UK10K haplotype panels.^15,16^ The imputation was carried out using the software IMPUTE2 resulting in data for 488 295 genotyped participants.^14,17^

### Quality Control

Participants were excluded from the study according to the following criteria: discrepancies between self-reported and genetically determined sex (data-field: 22001 and 31), a genotype missingness rate exceeding 0.05 (data-field: 22005), genetic relatedness (kinship coefficient greater than 0.0442), presence of sex chromosome aneuploidy (data-field: 22019) and anomalies in heterozygosity or missing rates (described in data-field: 22027, with heterozygosity over 0.1903). Only individuals of European descent were included, determined by self-reported ethnicity (data-field: 21000) and the exclusion of non-white backgrounds. This approach has been used successfully elsewhere.^11,18^

SNP exclusions were based on several criteria: Hardy-Weinberg equilibrium with a *P*-value smaller than 1E-10, a minor allele frequency (MAF) above 0.001, a missingness level exceeding 0.05, and an imputation score below 0.8. After exclusions were carried out in Python (v3.10), 13 380 287 SNPs remained. To link the SNPs to possible genes, we utilised eQTL datasets curated by the eQTL Catalog^19^ and GTEX consortium,^20^ focusing on data derived from bone/muscle/adipose/cartilage tissues and associations with *P* value less that 1E-05.

### Genomic Association Analyses

Exploratory data analyses were undertaken in both R (v4.3) and Python (v3.10). SNP based genetic association analysis of the dichotomous variable of hip fracture was carried out across the entire genome using PLINK. Age, sex and genetic principal components (PCs) were used as covariates (1-10 PCs).^21^ Genetic principal components were calculated by the curators of UK Biobank and provided as part of the UK Biobank dataset.^14^ Hip fractures were identified from hospital records using the following International Classification of Diseases (ICD) codes: 8200, 8202, 8208, S72.0, S72.00, S72.01.

## Results

**Figure 1.**
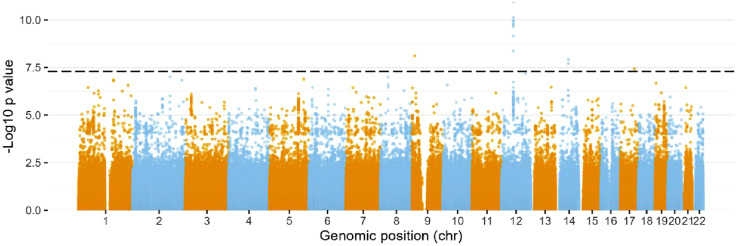
SNPs Associated with Neck of Femur Fracture in the UK Biobank. Manhattan Plot Displaying Chromosome Loci on the Horizontal Axis and the –log10(p) Values for Independence on the Vertical Axis, where Higher Vertical Points Suggest Stronger Associations. The Dotted Line Marks the Widely Accepted GWAS Threshold of 5E-8.

Through mining the UK biobank, 2165 neck of femur fractures were identified in the 502 507 participants. The mean age of hip fracture was 70 years in women and 67 years in men. To narrow down to the most likely biologically relevant SNPs, SNPs were filtered to those who had a MAF >0.001. 13 SNPs were identified as significantly associating with hip fracture using the stringent GWAS threshold of 5E-8 (Figure 1).

Eight SNPs are significantly associated with the binary variable of neck of femur fracture across four different loci described by the 1000 genomes project from the GWAS analysis (Table 1a). The most significant lead SNP associated with neck of femur fracture within the recognised haplotype was rs11614913[T] (*P* = 1.06E-10, beta = 0.228). This SNP is located on chromosome 12 (12q13.13). This association validates a previous observation of rs11614913[T] being associated with an increased risk of hip fractures (*P* = 1.0 × 10^−8^, OR = 1.11), which was described at the time as the “first sequence variant reported to associate with hip fractures”.^22,23^ Further, rs11614913[T] has previously been associated with waist circumference adjusted for body mass index^24,25^ (*P* = 4 × 10^-8^ & 8 × 10^−11^), spine bone size^22,23^ (*P* = 2 × 10^−42^) and bone mineral density in the neck of femur and lumbar spine in women^23^ (*P*-value = 9.9 × 10^−7^ and *P*-value = 3.2 × 10^−11^ respectively). Here we see the association with neck of femur fracture with sex as a covariate in the linear model, suggesting the effect on hip fractures, unlike the previously described effect on bone mineral density, is independent of sex. Other SNPs in this locus include rs56154542[C], rs56368105[G] and rs3803042[A] which are associated with waist-to-hip ratio adjusted for BMI & waist-hip index in a different study.^26^ The most severe consequence of rs11614913[T] has been to affect MIR196A2 and its microRNA transcript.^27^

**Table 1.** SNPs Associated with Neck of Femur Fracture in the UK BIOBANK (MAF > 0.001) Part (a) Displays SNPs and Recognised Haplotypes as Described in the 1000 Genomes Project, Whereas Part (b) Shows Several SNPs in a Putative Haplotype that is also Associated with Neck of Femur Fracture.

rsID	Chr	Position	REF	Alt/Minor	MAF (4 d.p)	*P*	beta (2 d.p)
A							
rs575603778	9	11184749	C	A	0.0011	7.70E-09	1.58
rs56154542	12	54393783	G	C	0.4070	1.10E-10	0.23
rs11614913^†^	12	54385599	C	T	0.4165	1.06E-10	0.23
rs56368105	12	54393770	A	G	0.4257	2.21E-10	0.22
rs3803042	12	54387947	G	A	0.4271	1.78E-10	0.22
rs538670058^†^	14	59140844	C	T	0.0026	1.20E-08	1.16
rs561165601	14	59163940	T	C	0.0027	1.94E-08	1.14
rs143842828	17	63367490	G	C	0.0012	3.70E-08	1.49

rsID	Chr	Position	REF	Alt/Minor	MAF (4 d.p)	*P*	beta (2 d.p)
B							
rs375577652	12	54 386 598	GGA	G	0.3305	4.30E-09	0.23
rs4142680^†^	12	54 405 190	C	T	0.4004	1.13E-11	0.25
12:54402808_GCGC_G	12	54402808	GCGC	G	0.4108	7.35E-11	0.23
rs200057925	12	54410007	C	CCCCTAG	0.4130	6.89E-10	0.22
rs138542768	12	54390749	G	GAAAAT	0.4251	1.56E-10	0.22

Previous work has shown rs11614913[T] induces a wobble bond in the primary miR-196a-2 hairpin transcript, changing the pairing from U to G instead of the usual C to G, leaving the seed sequences of both the 5p and 3p strands of duplex unaffected.^22^ Thus, the variant might affect miR-196a-5p target gene suppression by affecting the thermodynamic stability or processing of the mature miR-196a-5p duplex.^23,28,29^

In trying to understand further if rs11614913[T] may influence miR-196a-5p, we turned to other fields. Work in the human embryonic kidney cell line (HEK293) showed the rs11614913[T] suppressed genes significantly influenced gene expression, being less effective in suppressing miR-196a-5p target genes.^22^ Further, analysis of breast tumours showed samples homozygous for CC of rs11614913 trended towards having a higher fold increase of mir-196a (3.21 ± 1.00 [CC], 1.19 ± 0.24 [TT], *P* = 0.075).^30^ A study of non-small cell lung cancer tumours showed that homozygous mutation of rs11614913 [CC] was associated with a statistically significant increase in mature hsa-mir-196a expression but not with changes in levels of the precursor, suggesting enhanced processing of the pre-miRNA to its mature form. Furthermore, binding assays revealed that the rs11614913 SNP can affect binding of mature mir-196a-3p to its target mRNA. Survival was significantly decreased in individuals who were homozygous.^31^ Thus reduction in miR-196a-5p levels is likely driven by rs11614913[T], which has been shown to modulate the expression of homeobox C8 (HOXC8) across different tissues.^22,23^

A SNP in a different haplotype, rs4142680[T] is found at the same genomic region as HOXC8, which has been described as affecting the 3’ UTR. This SNP has been described in previous work as associated with hip fracture and decreased femoral neck bone mineral density in a study which included 11 516 hip fracture cases and 723 838 controls from the the Trøndelag Health Study (HUNT, Norway), Umeå Fracture and Osteoporosis Study (UFO, Sweden), UK Biobank, Estonian Biobank, and FinnGen biobanks (Finland).^32^ This result underlines how genetic polymorphisms affecting HOXC8 seem to influence hip fracture risk in the general population. Both rs11614913[T] and rs4142680[T] were found to be expression quantitative trait loci (eSNPs) for other homeobox-C cluster genes, including homeobox C6 (skin), homeobox C9 (adrenal gland, lung, tibial artery, tibial nerve), and HOXC Cluster Antisense RNA 1 (HOXC-AS1). However, current literature on their involvement in biological processes related to bone development is sparse.

Other loci associated with hip fractures were found on chromosome 9 with lead SNP rs575603778[A] which is an Intron Variant of LOC102724027 (9p21.1). Little is known about this RNA gene and it has previously not been associated with traits in the GWAS catolog. Two SNPs from the other associating locus, rs538670058[T] and rs561165601[C] have no reported gene consequence as does rs143842828[C], found in a separate haplotype.^33^

## Discussion

Our study highlights SNPs affecting the homeobox-C cluster genes influence hip fracture risk in the general population (HOXC6, HOXC8, HOXC9, and HOXC-AS1). The HOX-C cluster has been shown to influence skeletal development,^32^ with HOXC8 appearing to be particularly important.

HOXC8 has been shown to be important in murine skeletal development, with correct temporal expression of HOXC8 being key for determining the correct identity of the vertebral column in early embryos.^34^ Aberrant expression of HOXC8 is also associated with homeotic transformation of thoracic vertebrae.^35,36^ The tissue-specific overexpression of HOXC8 has been shown to inhibit chondrocyte maturation and stimulate chondrocyte proliferation.^36^ HOXC8 has been described as a major mechanism of osteoblast differentiation in bone morphogenetic protein (BMP) induced skeletal development, thus anything impeding its function even slightly may influence osteoblastic activity, bone development and fracture risk.^37,38^

Whilst this study specifically focuses on hip fractures, we acknowledge that SNPs associated with fracture risk may also contribute to fractures in other skeletal regions. Future studies should explore whether individuals carrying these SNPs exhibit an increased overall fracture risk beyond the femoral neck. Additionally, the UK Biobank does not provide data on femoral neck shaft angle, femoral physiological curvature radius, or bone density for all participants. Future research should consider incorporating these structural parameters, as well as gait analysis, to better understand the biomechanical impact of homeobox-C cluster gene variants on fracture susceptibility.

The effect of SNPs on HOXC8 has only been shown to influence fracture risk in European populations to date, which is one of the major limitations of many GWAS cohorts.^39-42^ This population is narrower, limited to those residing in the UK during recruitment. Further the participants do have a lower mean age of fracture than is typical for the UK (∼77 years), perhaps highlighting a recruitment bias towards more active individuals. Another limitation is that some samples were removed following the quality control covered in methods, therefore perhaps losing valuable cases. Further, it should be noted that this GWAS represents a snapshot in time. This consideration applies to all genome-wide studies where ongoing data collection may influence future findings.

Currently, the detailed characterisation of fractures in individuals with SNPs influencing the HOX-C cluster remains unclear. It is possible that aberrant expression of genes within this cluster may predispose individuals to specific fracture patterns in the hip and other bones. Additionally, whether screening for HOX-C-affecting SNPs in European populations could offer clinical benefit and whether traditional fracture prevention strategies remain effective in SNP carriers require further investigation. While this work provides a foundation for exploring personalised hip fracture prevention, further validation studies and clinical research are necessary to fully assess its implications.

## ORCID iD

Louis John Koizia https://orcid.org/0000-0003-4074-6926

## Statements and Declarations

### Ethical Approval

Approval was received from the UK Biobank (application number 79840). The UK Biobank secured approval from the National Research Ethics Service Committee.

## Data Availability

The datasets generated and/or analysed during the current study are not publicly available because the data for this study was made available under an application to the UK Biobank consortium. We are not at liberty to make their data public. Access is controlled and researchers can apply for this data directly from the UK Biobank consortium. Data are available through an application to the UK Biobank (https://www.ukbiobank.ac.uk/) and also are available from the corresponding author with blessing from the UK Biobank consortium on request.
